# Maternal Stress and Coping Strategies in Developmental Dyslexia: An Italian Multicenter Study

**DOI:** 10.3389/fpsyt.2017.00295

**Published:** 2017-12-22

**Authors:** Marco Carotenuto, Antonietta Messina, Vincenzo Monda, Francesco Precenzano, Diego Iacono, Alberto Verrotti, Alessandra Piccorossi, Beatrice Gallai, Michele Roccella, Lucia Parisi, Agata Maltese, Francesco Lavano, Rosa Marotta, Serena Marianna Lavano, Valentina Lanzara, Roberta Ida Ferrentino, Simone Pisano, Margherita Salerno, Anna Valenzano, Antonio Ivano Triggiani, Anna N. Polito, Giuseppe Cibelli, Marcellino Monda, Giovanni Messina, Maria Ruberto, Maria Esposito

**Affiliations:** ^1^Clinic of Child and Adolescent Neuropsychiatry, Department of Mental Health, Physical and Preventive Medicine, Università degli Studi della Campania “Luigi Vanvitelli”, Naples, Italy; ^2^Department of Experimental Medicine, Section of Human Physiology, Unit of Dietetics and Sports Medicine, Università degli Studi della Campania “Luigi Vanvitelli”, Naples, Italy; ^3^Brain Development Laboratory, Biomedical Research Institute of New Jersey, BRInj, Cedar Knolls, NJ, United States; ^4^Department of Pediatrics, University of L’Aquila, Ospedale San Salvatore, L’Aquila, Italy; ^5^Unit of Child and Adolescent Neuropsychiatry, University of Perugia, Perugia, Italy; ^6^Child Neuropsychiatry, Department of Psychology and Pedagogical Sciences, University of Palermo, Palermo, Italy; ^7^Department of Medical and Surgical Science, University “Magna Graecia”, Catanzaro, Italy; ^8^Department of Health Sciences, University “Magna Graecia”, Catanzaro, Italy; ^9^Department of Clinical and Experimental Medicine, University of Foggia, Foggia, Italy; ^10^Complex Structure of Neuropsychiatry Childhood-Adolescence of Ospedali Riuniti of Foggia, Foggia, Italy; ^11^Department of Medical-Surgical and Dental Specialties, Università degli Studi della Campania “Luigi Vanvitelli”, Naples, Italy

**Keywords:** children, parental stress, maternal emotions, developmental dyslexia, coping strategies

## Abstract

**Background:**

Studies about the impact of developmental dyslexia (DD) on parenting are scarce. Our investigation aimed to assess maternal stress levels and mothers’ copying styles in a population of dyslexic children.

**Methods:**

A total of 874 children (500 boys, 374 girls; mean age 8.32 ± 2.33 years) affected by DD was included in the study. A total of 1,421 typically developing children (789 boys, 632 girls; mean age 8.25 ± 3.19 years) were recruited from local schools of participating Italian Regions (Abruzzo, Calabria, Campania, Puglia, Umbria, Sicily) and used as control-children group. All mothers (of both DD and typically developing children) filled out an evaluation for parental stress (Parenting Stress Index—Short Form) and coping strategies [Coping Inventory for Stressful Situations (CISS)].

**Results:**

No statistical differences for mean age (*p* = 0.456) and gender (*p* = 0.577) were found between DD and control children. Mothers of children affected by DD showed an higher rate of all parental stress indexes (Parental Distress domain *p* < 0.001, Difficult Child *p* < 0.001, Parent–Child Dysfunctional Interaction *p* < 0.001, and Total Stress subscale score *p* < 0.001) than controls mothers. According to the CISS evaluation, mothers of DD children reported a significantly higher rate of emotion-oriented (*p* < 0.001) and avoidance-oriented (*p* < 0.001) coping styles than mothers of typical developing children. On the other hand, a lower representation of task-oriented coping style was found in mothers of DD children (*p* < 0.001) in comparison to mothers of control-children.

**Conclusion:**

Our study shows the clinical relevance of the burden carried by the mothers of children affected by DD and suggests the importance to assess parents, particularly mothers, to improve family compliance and clinical management of this disorder.

## Introduction

Developmental dyslexia (DD) is commonly identified only by reading difficulties, however, it should be considered a disability impacting multiple aspects of the life, particularly during pediatric age (1). In fact, DD is a complex neurodevelopmental deficit characterized by impaired reading acquisition despite the presence of adequate neurological and sensorial conditions, educational opportunities and normal cognitive level (2). Different cognitive and behavioral aspects are impaired in DD children such as sleep regulation (3), postural control (4), dental occlusion (5), mood regulation (6), and self-esteem (7). In this light, family support may be considered essential, particularly during the transition to adolescence and adulthood (8), and certainly relevant in pediatric age due to the natural frailty of this crucial period of life. Academic problems are related to a wide range of psychosocial problems, such as inattentiveness, low motivation for schoolwork, dropping out of school, fear of failure, depression, anxiety, loneliness, low self-esteem, and poor peer relations (9). Children affected by DD, as children with other specific learning disabilities, are also at greater risk of being bullied by their peers (9, 10). In 1996, Forness and Kavale (11) reported findings from a meta-analysis study on 152 studies about the nature of social skill deficits among learning disabled students. According to the teachers’ perception, children with learning disabilities tend to manifest socially withdrawn behavior and increased levels of hyperactivity and distractibility, but when evaluated by their peers, they appeared to be defined primarily by their reduced acceptance and greater rejection. Social dysfunction could be caused by different types of variables (e.g., congenital deficits, neuropathologic abnormalities, language disorders, memory impairment, cognition delays, preterm birth, etc.), which may contribute to determine academic problems.

Specifically, preterm birth (12–15), prenatal insults and maternal stress during pregnancy (16–18), prenatal exposure to nicotine (19) may be considered as relevant for generic reading difficulties and also for dyslexia. In general, also the attachment and bonding process should be considered as mandatory to threat in order to promote the parental well-being and in order to minimize the morbidity of preterm birth such as reading problems (20, 21).

Independently from the risk factors for reading disabilities, family support is essential for coping strategies, considering that parenting, may be conceptualized in terms of two orthogonal dimensions of demandingness and responsiveness. Generally, the parenting styles were originally conceptualized as transactionally associated with social competence, but studies have mostly focused on parent-to-child effects. In this perspective, adolescent behavior had a much stronger effect on parenting styles than the reverse, while significant child effects were found for permissive-indulgent parenting (22). About parental coping skills, parents’ perceptions of their child’s illness are based on the knowledge that was already in their possession prior to its onset and on the information that they are either provided with or actively seek out from professionals, or from informal sources, after receiving their child’s diagnosis. These mental representations of the illness are related to the way these parents process and cope with their knowledge of their child’s illness. In general, two ways to cope are recognized with threatening information: monitoring and blunting, where monitoring is expressed by seeking threat-relevant information, and blunting by avoiding (23).

On the other hand, Kavale and Fornes spin pointed out lack of self-esteem among students with learning disabilities, with a general feelings of inferiority (11), which is often evidenced by the use of compensatory learning instruments such as audiobooks or playing different academic activities in comparison to their peers (24).

Independently on daily difficulties of DD children, familiar background is not well identified and usually not evaluated in the clinical practice. On the other terms, illness acceptance may be considered as relevant, particularly when health problem can impact the daily life functioning. In this framework, the aim of this multicenter study was to evaluate the impact of DD children on maternal coping styles and stress management.

## Materials and Methods

### Study Population

The study population of this Italian multicenter study comprised a total of 874 children (500 boys, 374 girls) with a mean age of 8.32 ± 2.33years diagnosed with DD and consecutively referred to the all pediatric participants centers, according to ICD-10 criteria (25). In order to compare all data, a total of 1,421 typically developing children (789 boys, 632 girls) with mean age 8.25 ± 3.19 years was recruited from local schools of participating Italian Regions (Abruzzo, Calabria, Campania, Puglia, Umbria, Sicily) (Table 1). The protocol study was approved by local University Ethics Committee. The study was conducted according to the ethical standards of 1964 Helsinki Declaration and its later amendments or comparable ethical standards. Written informed consent was obtained from all parents of the pediatric patients. The two groups were comparable for socioeconomic status and educational level, assessed with according to the Hollingshead Four Factor Index of Social Status (26).

**Table 1:** Flow chart.

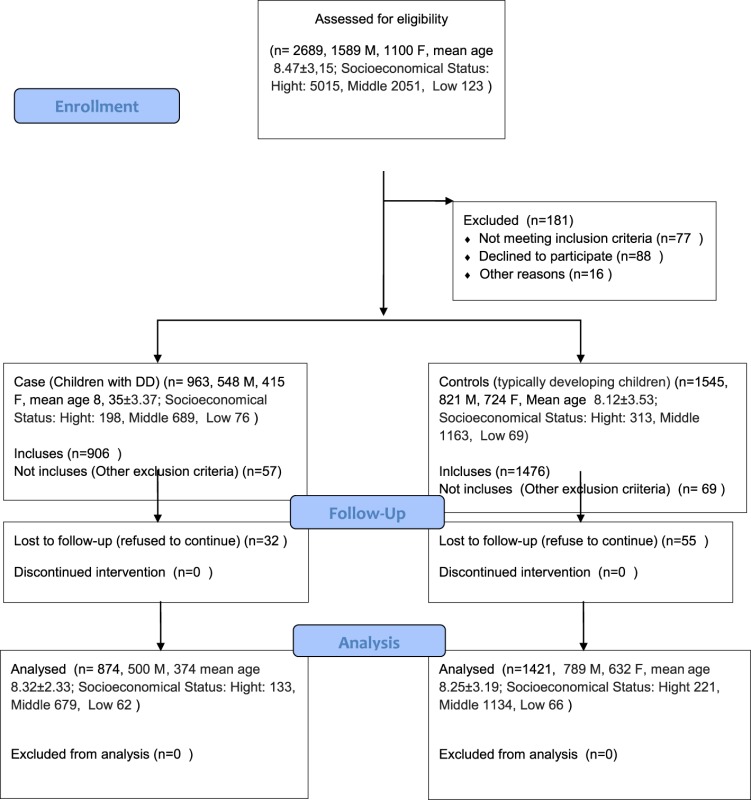

Exclusion criteria were: neurological disorders (i.e., epilepsy, neuromuscular disorders, cerebral palsy), psychiatric symptoms (such as Attention-Deficit/Hyperactivity Disorder, internalizing, and externalizing problems); intellectual disability (IQ ≤ 70); borderline intellectual functioning (IQ ranging from 71–84).

### Cognitive Screening

The nonverbal intelligence level was assessed using the Raven Coloured Progressive Matrices test for a quick cognitive screening (27). Each of the 36 test items consists of an incomplete abstract pattern. Participants are required to select, from a set of six, the figure needed to complete the pattern correctly. The raw scores were converted into *z*-points with reference to Italian normative data; thereafter, the *z*-points were converted into IQ scores. The reliability of the test is about 0.90.

### Reading Ability Assessment

Reading abilities were evaluated by means of word, pseudoword (28), and short story reading tests (29); these tests allowed us to establish, with reference to Italian normative data for every age group, each child’s reading fluency (number of syllables read per second, syll.s/sec) and reading accuracy (number of mistakes made) for each of these tasks (reading aloud), giving an overall total of six parameters. These are key parameters in transparent orthographies, like Italian. The reliability of the tests ranges from 0.752 to 0.869 for accuracy and from 0.943 to 0.967 for fluency. The results were considered poor if the parameter values were <1.5 SD (fluency) or <5th percentile (accuracy).

Reading comprehension was evaluated using Italian texts appropriate for the child’s age and school year and the evaluation consisted of silent reading followed by ten multiple-choice questions. One point was given for each correct answer (29). The reliability of the tests ranges from 0.573 to 0.700. A total score below the 25th percentile, according to Italian normative data, indicated the presence of a reading comprehension problem.

### Parenting Stress Index—Short Form (PSI-SF)

Accordingly to Esposito et al. (30), the perceived parental stress evaluation among mothers of both groups was performed with the Italian version of PSI-SF (5). The PSI-SF is a standardized too that yields scores for parental stress across four areas *via* Parental Distress (PD) and Parent–Child Dysfunctional Interaction (PCDI) domains and Difficult Child (DC), and Total Stress subscales. It has 36 items and provides both raw and percentile scores. Each item is graded on a five-point Likert scale, from 1 (strongly disagree) to 5 (strongly agree). The PD domain measures the distress that parents feel about their parenting role in light of other personal stresses and has a cutoff score of 36. The PCDI domain focuses on the perception of the child as not responsive to parental expectations, and has a cut-off score of 27. The DC subscale represents behaviors that children often engage in that may make parenting easier or more difficult, and has a cut-off score of 36. The PSI-SF also produces a Defensive Responding (DEF) subscale score, which indicates likely response bias. The subscale scores range from 12 to 60 and the Total Stress subscale scores ranges from 36 to 180, with higher scores indicating greater levels of parental stress. Thus, responses higher than the 85th percentile (1 SD above the mean) are interpreted as clinically significant for high levels of family stress (5). The PSI-SF has been used widely, and psychometric evidence supports its reliability and validity. The PSI-SF shows high internal consistency (Cronbach’s alpha 0.92) and its validity has been established in parents of children with chronic medical conditions, including diabetes and asthma (5, 31). In this study, the PSI-SF was administered only to the mother, being the parent assumed to usually spend more time with the children.

### Coping Inventory for Stressful Situations (CISS)

As reported by Iavarone et al. (32), the Italian version of CISS was widely used to assess the parental coping strategies (33). The CISS is a 48-item self-report and has been developed to describe cognitive styles and behavioral resources in response to a specific stressor (34). It assesses three coping strategies:

– task-oriented coping (16 items), which refers to purposeful efforts aimed at solving and/or restructuring the problem in an attempt to improve the situation;
– emotion-oriented coping (16 items), which refers to self-oriented reactions including emotional responses, self-preoccupation, and fantasizing;
– avoidance-oriented coping (16 items), which refers to activities and cognitive changes aimed at avoiding the stressful situation by distracting oneself with other situations or tasks, or *via* social diversion as a means of alleviating stress (34).

Each item ranges from 1 to 5 (1 rates as “not at all” and 5 rates as “very much”). Subjects are asked to think about a variety of stressful and upsetting situations and the rating scales are used to indicate how often the respondent engages in the behaviors presented, which is how the range of 1–5 is used. In order to compare the results of each coping strategy scale the standard points were used for this study (32–34).

### Statistical Analysis

The *t*-test for unpaired samples and chi-square test were applied, when appropriate, to compare demographic characteristics (age, gender), PSI-SF, and CISS results between DD vs. control children populations. We used the *t*-test as the groups were not different confounding factors (namely age and gender all *p* > 0.05). We accounted for multiple comparisons by using a Bonferroni correction. In particular, we divided *p*-values for the number of comparisons (35) and set our threshold for a significant *p*-value below 0.001. Therefore, were considered significant only *p*-values lower than 0.001. All data were coded and analyzed using the commercially available STATISTICA package for Windows (v 6.0; StatSoft Inc., Tulsa, OK, USA).

## Results

No statistical differences were found between DD vs. control group for mean age (*p* = 0.456) and gender (*p* = 0.577). The parental stress examination in mothers of children affected by DD showed an higher rate of all parental stress indexes, specifically they reported higher mean on the PD domain (26.78 ± 8.74 vs. 16.87 ± 6.13; *p* < 0.001), DC subscale (31.58 ± 8.79 vs. 28.66 ± 3.48; *p* < 0.001), PCDI domain (21.43 ± 7.99 vs. 20.16 ± 2.96; *p* < 0.001), and Total Stress subscale score (87.13 ± 14.79 vs. 69.89 ± 13.54; *p* < 0.001) than the mothers of typically developing children, as shown in Table 2. No relevant differences between the two groups were found for the DEF domain scores (17.25 ± 4.32 vs. 17.54 ± 4.81; *p* = 0.141) (Table 2).

**Table 2:** Comparison of PSI-SF results between mothers of dyslexic children and mothers of typical developing children.

	DD, *n* = 874	Controls, *n* = 1,421	*t*-Value	95%IC	*p*	Hedges’ *g* effect size
PD	26.78 ± 8.74	16.87 ± 6.13	31.86	9.30–10.52	<0.001	1.37
PCDI	21.43 ± 7.99	20.16 ± 2.96	5.42	0.81–1.73	<0.001	0.23
DC	31.58 ± 8.79	28.66 ± 3.48	11.18	2.41–3.43	<0.001	0.48
DEF	17.25 ± 4.32	17.54 ± 4.81	1.46	−0.68–0.10	0.1452	0.06
Total stress	87.13 ± 14.79	69.89 ± 13.54	28.59	16.06–18.42	<0.001	1.23

*The mean differences among children affected by developmental dyslexia (DD) and typical developing children (Controls) in Parenting Stress Index—Short Form (PSISF) scales: Parental Distress (PD); Parent–Child Dysfunctional Interaction (PCDI); Difficult Child (DC); Defensive Responding (DEF). *t*-Test was applied. *p*-Values < 0.05 were considered statistically significant. Hedges’ *g* analysis was performed in order to calculate the effect size weighted according to the relative size of each sample*.

According to the CISS evaluation, mothers of DD children reported a significantly higher rate of emotion-oriented (71.43 ± 7.45 vs. 52.13 ± 6.12; *p* < 0.001) and avoidance-oriented (68.15 ± 6.33 vs. 52.61 ± 5.51; *p* < 0.001) coping styles than mothers of typical developing children. On the other hand, a lower representation of task-oriented coping style was found in mother of DD children (14.57 ± 8.29 vs. 65.86 ± 5.81; *p* < 0.001) than in mothers of controls (Table 3).

**Table 3:** Comparison of coping strategies between mothers of dyslexic children and Controls.

	DD, *n* = 874	Controls, *n* = 1,421	*t*-Test value	95%IC	*p*	Hedges’ *g* effect size
Task-oriented	14.57 ± 8.29	65.86 ± 5.81	173.9104	−51.87–50.71	<0.001	7.47591
Emotion-oriented	71.43 ± 7.45	52.13 ± 6.12	67.4358	18.74–19.86	<0.001	2.898874
Avoidance-oriented	68.15 ± 6.33	52.61 ± 5.51	61.9459	15.05–16.03	<0.001	2.662877

*Differences among mothers of children affected by developmental dyslexia (DD) and mothers of typical developing children (controls) in Coping Inventory for Stressful Situations (CISS). *t*-Test was applied. *p*-Values < 0.05 were considered statistically significant. Hedges’ *g* analysis was performed in order to calculate the effect size weighted according to the relative size of each sample*.

For both Tables 2 and 3, Hedges’ *g* Effect size was calculated.

## Discussion

Higher levels of stress rate were found in mothers of children affected by DD respect of healthy children. Particularly, mothers of DD children showed higher scores in all domains of PSI-SF such as PD, DC, and PCDI subscales than mothers of typically developing children, suggesting that mothers of DD children seem to consider as stressors each interaction with their own children. In general, learning difficulties and/or scholastic problems tend to impact negatively on parenting quality due to the high level of stress, as showed by Loprieno et al. (36) when assessing parents of children with ADHD. In general, children with learning disabilities tend to present lower self-concept, more anxiety, and lower peer acceptance than peers. Meanwhile, the invisible disability may create intolerance toward the child by the family and general public (37). Moreover, learning disabilities may generate false hope in parents (38), who may initially respond to the diagnosis with denial of, and ambivalence about, the child’s disability and unrealistic expectations for his or her academic performance (39). These conditions would heighten parental stress and cultivate negative family functioning (40). Alternatively, considering that among DD the mediational role of family support is relevant, obviously parents’ worrying seems to be dependent on their coping strategies and be crucial to balance the peer refusal in all ages, comprising adulthood (41). On the other hand, having a child with learning disorders appears to predispose parents to higher levels of frustration and dissatisfaction. In fact, mothers who reported high levels of stress from these life events appear to be more controlling, abusive and punitive than mothers who have lower levels of stress (42, 43). Moreover, the additional stress associated with raising a child with learning disabilities may affect children in several ways including insecure attachments of the child to the parents (44), low family cohesion (26), and increases in both internalizing and externalizing behavior problems (43). Again, children with learning disabilities are more dependent on the others (e.g., adults) and often lag behind their peers in terms of their level of independence (44). This dependence may also predispose their parents to higher levels of stress (43) as showed by findings of present study. In particular, mothers with DD children present a higher rates of emotion-oriented and avoidance-oriented coping styles that include higher rates of self-oriented reactions (i.e., emotional responses, self-preoccupation, and fantasizing), or the presence of activities and cognitive changes aimed to avoid the stressful situation by distracting themselves with other situations or tasks, or *via* social diversion as a mean to alleviate the stress than mothers of healthy children. Moreover, mothers of DD children present lower rates of task-oriented coping strategies including purposeful efforts aimed at solving and/or restructuring the problem in an attempt to improve the situation. This style could be considered more useful for the DD management and stress reduction in mothers of DD children and seems to justify the clinical improvement of the parental stress in these mothers. The results suggest the need of support families who have a child with a learning disability, and particularly affected by DD. In this light, school programs and procedures for identification and placement of these children may be reexamined. Earlier and identification of a child’s difficulty followed by appropriate educational placement would be necessary to satisfy parents and reduce their stress. This however, cannot be possible without adequate funds. Educational programs should develop social and behavioral competence in children with learning disabilities (39). Moreover, we have also to consider the role of other conditions that can impact the reading abilities during childhood and adolescence such as rolandic epilepsy (45), temporo-occipital epilepsy, frontal lobe epilepsies, Panayiotopoulos syndrome, benign epilepsy with centrotemporal spikes and daytime seizures, the use of antiepileptic drugs, and interictal discharges (46) because of their direct effect on cognitive abilities. Similarly, neuromuscular disorders such as Duchenne muscular dystrophy may reduce the academic and reading ability for impairments in phonological processing and rapid lexical access (47) and so acts the borderline intellectual functioning (35, 48).

Regarding the role of ADHD, this condition may impact the reading capacity for impairing on complex bimanual out-of-phase movements and with manual dexterity (49, 50) similarly to depression and anxiety that are more frequent in dyslexic subjects (51–53).

In conclusion, our study highlighted a new aspect of this multifaceted disease as DD, suggesting the relevance of caregivers styling evaluation for an adequate clinical management and to improve the family compliance.

## Ethics Statement

The protocol study was approved by local University Ethics Committee. The study was conducted according to the ethical standards of 1964 Helsinki Declaration and its later amendments or comparable ethical standards. Written informed consent was obtained from all parents of the pediatric patients.

## Authors Contributions

All authors contributed to patients’ recruitment, data collection, and processing. All authors participated to drawing up the manuscript and were involved to the intellectual workup for the article. All authors read and approved the final manuscript.

## Conflict of Interest Statement

The authors declare that the research was conducted in the absence of any commercial or financial relationships that could be construed as a potential conflict of interest.

## Abbreviations

CISS, Coping Inventory for Stressful Situations; DD, developmental dyslexia; PSI-SF, Parenting Stress Index—Short Form.
